# Evaluation of Apelin and Apelin Receptor Level in the Primary Tumor and Serum of Colorectal Cancer Patients

**DOI:** 10.3390/jcm8101513

**Published:** 2019-09-20

**Authors:** Marta Podgórska, Dorota Diakowska, Katarzyna Pietraszek-Gremplewicz, Miroslaw Nienartowicz, Dorota Nowak

**Affiliations:** 1 Department of Cell Pathology, Faculty of Biotechnology, University of Wroclaw, 50-383 Wroclaw, Poland; marta.podgorska2@uwr.edu.pl (M.P.); katarzyna.pietraszek-gremplewicz@uwr.edu.pl (K.P.-G.); 2 Department of Nervous System Diseases, Wroclaw Medical University, 51-618 Wroclaw, Poland; dorota.diakowska@umed.wroc.pl; 3 Department of Gastrointestinal and General Surgery, Wroclaw Medical University, 50-368 Wroclaw, Poland; miroslaw.nienartowicz@umed.wroc.pl

**Keywords:** adipokines, apelin, apelin receptor, colorectal cancer

## Abstract

Colorectal cancer is the second deadliest tumor, which has a positive correlation with obesity which led to increasing interest in the relationship between adipokines and cancer progression. Apelin is a secreted peptide involved in regulation of tumor progression and invasiveness. In this study, we examined apelin and apelin receptor expression level in colorectal cancer. Apelin, and its receptor mRNA, and protein expression levels were measured in tumor tissue of 56 surgically treated colorectal adenocarcinoma (CRC) patients. We also analyzed apelin and apelin receptor protein levels in sera of 56 CRC patients and 27 healthy controls. The mRNA and protein level of this peptide and its receptor was higher in tumors than that in control tissue. Serum levels of apelin and apelin receptor were increased in CRC patients in comparison to controls. The concentration of serum apelin level significantly increased in individuals with lymph node and distant metastasis. Obtained results suggest that apelin could be an important factor in progression of colorectal carcinoma.

## 1. Introduction

Colorectal adenocarcinoma (CRC) is the third most common cancer diagnosis and second deadliest tumour for both males and females [1]. More than 41% of all colon cancer cases occur in the proximal colon, whereas approximately 22% occur in the distal colon and 28% involve the rectum. Approximately 20% of patients diagnosed with CRC could develop metastasis. Most common directions of CRC metastasis include regional lymph nodes, lung, liver and peritoneum [2]. Recent studies suggested a correlation between the development of colorectal cancer and obesity. Therefore, adipokines-cytokines released by the adipose tissue could play an important role in the progression of this type of cancer [3]. 

Adipokines display many properties, including the regulation of blood pressure, carbohydrates and lipid metabolism and angiogenesis [4]. Apelin, that belong to the family of adipokines, is secreted as a 77-prepropeptide, that could be cleaved producing a group of apelin peptides [5]. In cancer, apelin and its receptor (APJ) are involved in signalling pathways connected with migration and invasion leading to tumour growth and metastasis [6]. 

Several studies presented that apelin could be a promising biomarker to predict therapy response and prognosis of patients’ survival. Zuurbier et al. demonstrated correlation between high concentration of tissue apelin and non-response to bevacizumab therapy in patients with CRC [7]. In gastroesophageal cell carcinoma serum, the tissue level of apelin was higher than in healthy controls [8]. In gastric cancer individuals, apelin level was related to clinical features and prognosis. Patients characterized by high tumor apelin expression had a shorter overall survival period than those with low expression [9]. Similar results were obtained in non-small cell lung carcinoma (NSCLC) [10], bladder cancer [11], cholangiocarcinoma (CCA) [12], head and neck cancer [13], prostate cancer [14], ovarian cancer [15], breast cancer [16], oesophageal squamous cell carcinoma [17], multiple myeloma [18], and glioblastoma [19].

According to our knowledge, there is no study including quantitative analysis of apelin and its receptor expression in colorectal carcinoma patients. Therefore, in this study we examined mRNA and the protein level of apelin and its receptor in CRC tumour tissue obtained from surgically treated patients. Considering the diagnostic potential of serum, as well as novelty of these data, we also analyzed apelin serum levels in patients diagnosed with CRC and healthy individuals. 

## 2. Materials and Methods 

### 2.1. Patient Characteristics

The study population consisted of 56 individuals (32 men and 24 women, with a mean age of 68.4 ± 11.1 years) with histologically confirmed colorectal cancer, admitted to the Department of Gastrointestinal and General Surgery, Wroclaw Medical University for curative resection of a tumor. Patients who underwent radio-or chemo-therapy prior surgery and patients with severe systemic illness were excluded from the study. Preoperative evaluation included blood diagnostics, physical examination and imaging techniques (such as ultrasonography, computed tomography, and magnetic resonance). 

Cancers were staged pathologically using the seventh edition of the Union for International Cancer Control (UICC) TNM Classification of Malignant Tumors (TNM) [20]. There were 17 patients with stage I, 17 with stage II, 13 with stage III, and 9 patients with stage IV cancer progression. According to the grading system, 53 tumours demonstrated moderate differentiation (G2) and 3 tumours showed poor differentiation (G3). In 20 patients, the tumour was located on the right colon, in 33 on the left colon, and in 3 patients on the rectum. The patients’ demographical, clinical and pathological characteristics are shown in Table 1.

Blood samples were taken from the CRC patients prior to any treatment. Samples of the tumour and matched non-tumour tissue were obtained intraoperatively and they were collected for the analysis of mRNA and protein level.

### 2.2. Ethical Consideration 

The study was conducted according to the ethical standards detailed in the Declaration of Helsinki, as revised in 1983. The study protocol was approved by the Medical Ethics Committee of Wroclaw Medical University (Poland). Informed consent was obtained from all subjects.

### 2.3. Tissue Extracts and Sera Isolation

The tissue samples were collected from the tumor and from the macroscopically normal tissue taken approximately 10 cm from the tumor, rinsed with phosphate buffered saline (PBS) and stored at −45 ℃ until examination. Prior to analysis, tissue samples were homogenized in 10 mM Tris-HCl with 150 mM KCl and 1 mM ethylenediaminetetraacetic acid (EDTA) pH 7.4 buffer (proportion 1:2 w/v) using a FastPrep-24 homogenizer (MP Biomedicals, Solon, OH, USA) for 2 min at 4.0 m/s. The homogenates were centrifuged at 14,500× *g* for 10 min at 6 ℃ and supernatants were used for apelin and apelin receptor determination.

The samples of peripheral blood were clotted (30 min, room temperature) and centrifuged at 1500× *g* for 10 min at room temperature. Obtained sera were stored at −45 ℃ until examination. 

### 2.4. Apelin Protein Level Determination

The concentrations of human apelin-36 and human apelin receptor in the tissue homogenates and sera were measured using commercially available enzyme-linked immunosorbent assay (ELISA) kits (apelin-36: MBS2021970, apelin receptor: MBS089535, MyBioSource Inc., San Diego, CA, USA) according to the manufacturer’s instructions. All measurements were performed in duplicate. The sensitivity of the apelin assay was 2.63 pg/mL and the apelin receptor assay was 1.0 ng/mL. Data for apelin levels were expressed as pg/mL of serum and ng/g of analyzed tissue, data for apelin receptor as ng/mL of serum and µg/g of tissue.

### 2.5. Transcript Level Quantification 

Total RNA was isolated using the phenol-chloroform method. The tissue samples for RNA extraction were homogenized with TRIzol Reagent (Thermo Fisher Scientific, Waltham, MA, USA) using a FastPrep-24 homogenizer (MP Biomedicals, Solon, OH, USA). RNA was quantified with a NanoDrop 2000 spectrophotometer (Thermo Fisher Scientific, Waltham, MA, USA). Its purity was evaluated by calculating 260/280 and 260/230 ratios. DNA was digested using a DNase I kit (EurX, Gdańsk, Poland).

Two micrograms of total RNA were reverse transcripted using a High-Capacity cDNA Reverse Transcription Kit (Thermo Fisher Scientific, Waltham, MA, USA). 

Apelin and apelin receptor mRNA expression levels were determined using a real-time PCR reaction. The qPCR reaction mixture contained 3 μL cDNA, 5 μL TaqMan™ Fast Universal PCR Master Mix (Thermo Fisher Scientific, Waltham, MA, USA), 0.5 μL TaqMan™ MGB Probe (glyceraldehyde 3-phosphate dehydrogenase (GAPDH): Hs02758991_g1, APLN: Hs00936329_m1, APLNR: Hs00270873_s1) (Thermo Fisher Scientific, Waltham, MA, USA) and 1.5 μL water. The reaction was conducted in triplicates using a StepOne Real-Time PCR System (Thermo Fisher Scientific, Waltham, MA, USA) with cycling parameters: 50 ℃ (2 min), 95 ℃ (10 min) and 40 cycles of 95 ℃ (15 s), and 60 ℃ (1 min). Cycle threshold (Ct) values were calculated using the StepOne Software v2.3 (Thermo Fisher Scientific, Waltham, MA, USA). A representative figure with the cycle curves is presented in Supplementary Figure S1. Apelin and apelin receptor genes expression levels were determined after normalization to GAPDH (glyceraldehyde 3-phosphate dehydrogenase) reference gene and calculated using the 2^(−ΔCt)^ method [21].

### 2.6. Statistical Analysis

All the data were analyzed using Statistica v. 13.0 software (StatSoft Inc., Tulsa, OK, USA). Data distributions were tested with the Shapiro–Wilk normality test and homogeneity of variances (HOV) were examined using the Brown–Forsythe test. Continuous variables were expressed as median (minimum–maximum) values. Paired samples were tested using the Wilcoxon test. Two independent samples were analysed with Mann–Whitney U test. More than two independent samples were compared using ANOVA analysis and the post-hoc Tuckey test or Kruskal–Wallis analysis of variance and the post-hoc Dunn test. Correlations between variables were tested with the Spearman correlation test for non-normally distributed variables. *p* values < 0.05 were considered to be significant.

## 3. Results

### 3.1. Comparison of Apelin and Apelin Receptor mRNA Expression Level between Tumour and Normal Tissue of CRC Patients

Analysis of mRNA expression showed increased levels of apelin in tumor tissue in comparison to non-tumor tissue in CRC individuals (*p* = 0.0007). The apelin tumour/non-tumour ratio showed 2.5 times higher levels of apelin in tumour tissue. The analogous result was obtained for apelin receptor expression level with *p* = 0.03 and a ratio of 4.24 (Table 2). 

### 3.2. Concentration of Apelin and APJ on Protein Level

**Figure 1:**
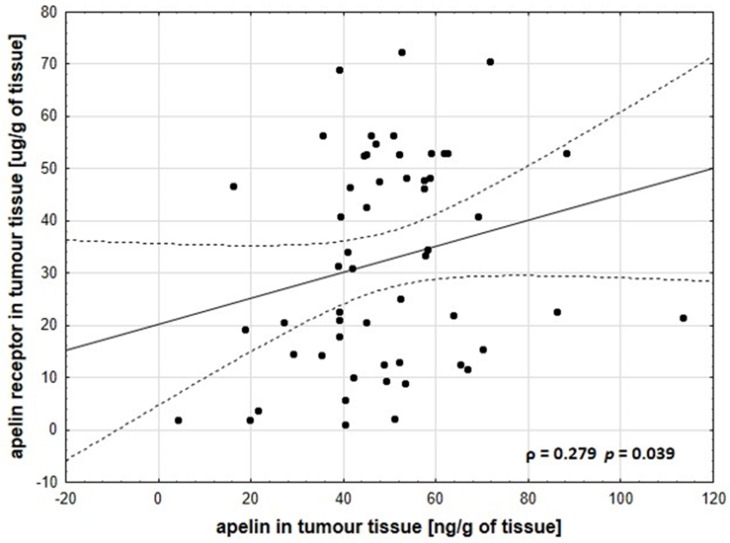
Positive correlation between apelin and its receptor levels in the tumour tissues of CRC patients. These data were analysed using the Spearman’s rank correlation coefficient (ρ).

Comparison of non-tumor and tumor tissue showed statistically increases of apelin concentration in CRC with *p* < 0.0001 and the apelin tumour/non-tumour ratio was 1.68. This phenomenon was also observed in the case of APJ level in CRC tissues (*p* < 0.0001, ratio = 7.98) (Table 3). Moreover, we observed a positive correlation between apelin and apelin receptor tumour tissue level with ρ = 0.279 and *p* = 0.039 (Figure 1). We did not observe correlation between tissue and the serum apelin level of CRC patients (*p* = 0.76) (Supplementary Figure S2). Interestingly, the relationship between clinical parameters and the concentration of apelin receptor in tumors showed statistically higher amounts of this protein in women diagnosed with CRC (*p* = 0.02) (Supplementary Figure S3). When the relationship between concentration of apelin in tumors and clinicopathological parameters was analysed, no differences were revealed (Supplementary Figures S4 and S5). 

### 3.3. Serum Levels of Apelin and Apelin Receptor in Cancer Patients

**Figure 2:**
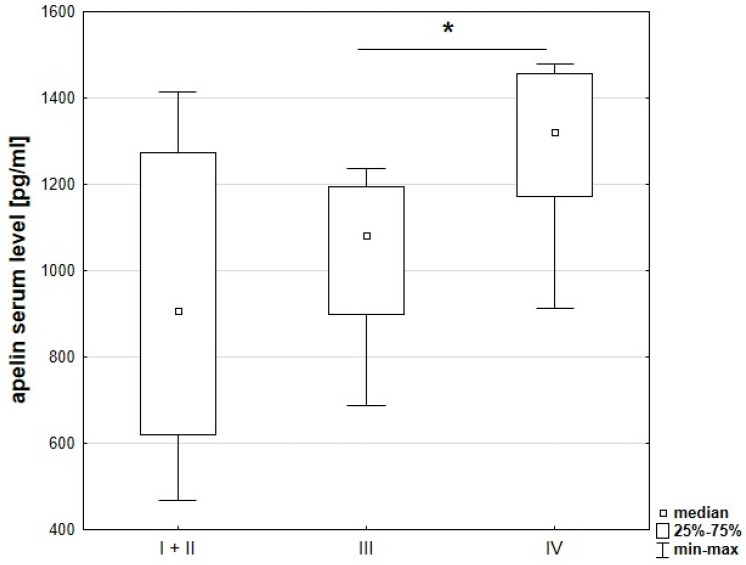
Apelin serum levels in patients with different stages of CRC. These data were analysed using the post-hoc Dunn’s test. * Statistically significant at *p* < 0.05.

**Figure 3:**
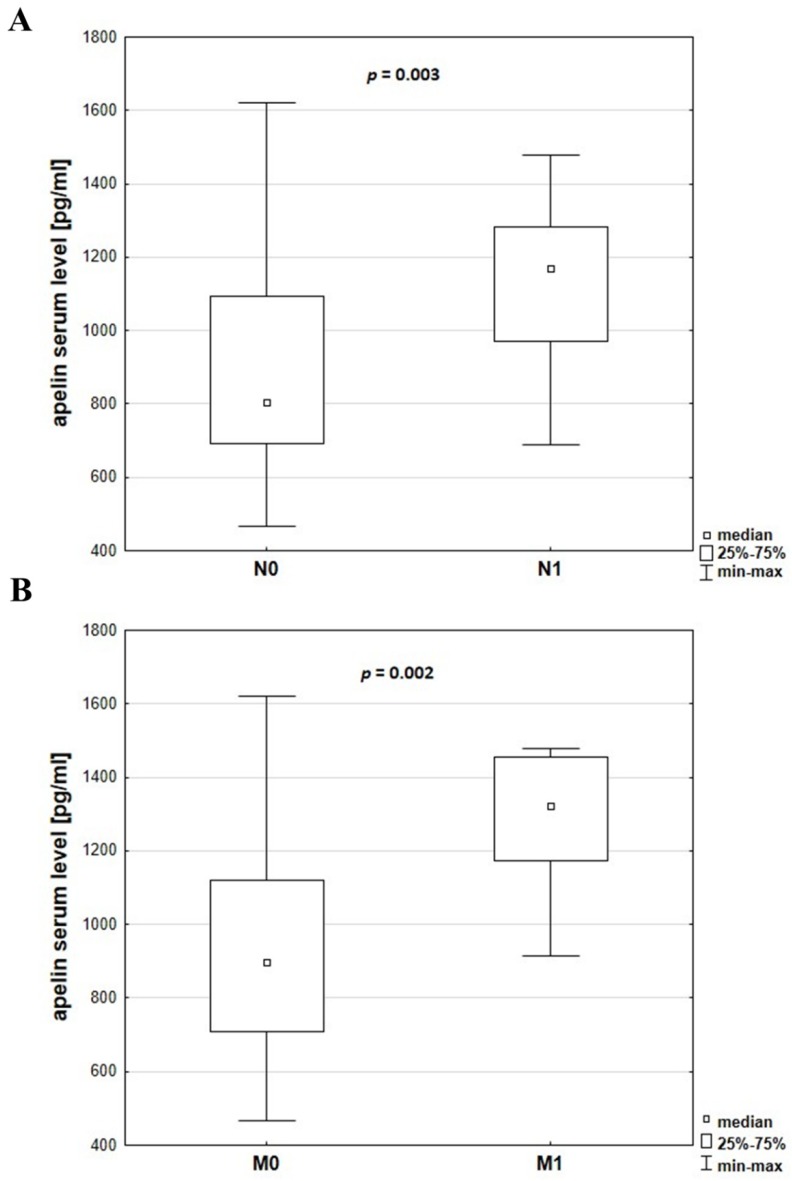
Apelin serum levels in CRC patients without (N0) or with (N1) lymph node metastasis (**A**) or without (M0) or with (M1) distant metastasis (**B**). The data were analysed using the Mann–Whitney U test.

Evaluation of cancer progression based on comparison of clinicopathological parameters and apelin serum levels showed a relationship between stage of cancer and apelin concentration. Level of serum apelin was significantly higher in patients at more advanced TNM stages (*p* = 0.021) (Figure 2). Increased levels of apelin were also observed in patients with lymph node metastasis (*p* = 0.003) (Figure 3A), as well as distant metastasis (*p* = 0.002) (Figure 3B).

Since we did not observe a positive correlation between tissue and serum apelin levels, we decided to analyze the influence of body mass index (BMI) on apelin plasma level according to data indicating that apelin serum level increases in obese patients [22]. Therefore, the correlation between plasma apelin concentration and BMI of CRC patients was examined. The analysis did not indicate differences in plasma apelin level (Supplementary Figure S6). 

## 4. Discussion

Apelin level was demonstrated to increase in colorectal carcinoma. This small adipokine is present in many organs, including kidney, liver, pancreas and brain [23]. In several types of cancer, it has been reported to regulate tumor growth, neoangiogenesis, cell migration and even metastasis induction. In non-small cell lung cancer, apelin was overexpressed in tumor tissue at the mRNA level, as well as at protein level detected by immunohistochemistry. Moreover, overexpression of the apelin gene in mice resulted in tumor growth, suggesting important involvement of this adipokine in the progression of NSCLC [10]. 

Our previous study showed that apelin added to culture medium stimulates proliferation, migration and invasion of colon cancer cell lines, connected with increased ability to migratory protrusions formation, metalloproteases secretion, and cytoskeleton rearrangement [24]. According to our knowledge, this is the first study focusing on mRNA and protein level of apelin and its receptor in primary colorectal carcinoma patients. We also detected apelin in patients’ serum, which is an important diagnostic parameter and could be useful in the determination of cancer risk factors. Similar studies were presented by Yang et al. who showed high apelin tumor concentration in patients with bladder cancer, which was closely associated with shorter overall survival and disease-free survival in muscle-invasive bladder cancer patients [11]. Hall et al. presented semi-quantitative analysis of CCA tissues that showed significantly increased expression levels of the apelin receptor compared to non-malignant tissue sections. The authors suggested, that tumor environment could promote upregulation of apelin and APJ in cholangiocarcinoma [12]. Moreover, in obese men with colon cancer, the level of serum apelin was increased in comparison to non-obese patients [25]. On the basis of these literature data, we hypothesised that level of apelin, as peptide released by adipose tissue, could be altered in colorectal carcinoma patients and correlate with CRC development. 

In our study, we presented the correlation of apelin and APJ mRNA level between tumur, and non-tumor tissue. Increased gene expression level stayed in line with the protein amount of tested adipokine. We also observed a link between apelin and its receptor concentration in tumor and non-tumor tissue. Increased apelin levels correlated also with apelin receptor levels in CRC tissue. These results parallel with other studies, in which apelin and APJ protein concentrations were increased in tumour tissue compared with normal tissue in oesophageal squamous cell carcinoma [17] or prostate cancer [14]. Interestingly, Yoo et al. in their correspondences did not observe the differences in the immunopositivity of apelin receptor between non-tumor and tumor tissue of CRC patients. This could be caused by the application of another method—immunohistochemistry, which is a rather semi-quantitative method. Moreover, the authors used polyclonal antibodies to determine if APLNR was present in CRC tissue. Additionally, there is no information concerning the technique of matching the samples [26]. The analysis of correlation between apelin tissue level and clinicopathological parameters revealed no significant differences. Conscious of the fact that examined patients’ tissues contained tumor cells, and also cancer environmental cells; in this stage of study, apelin could not be considered as a biomarker of CRC. However, the data obtained in this study are valuable and worth continuing with. 

Apelin is a secreted peptide that could circulate in serum, which is an easily accessible diagnostic material [6]. In our study, serum apelin concentration tended to be higher in patients with more advance TNM stages. Patients with lymph node and distant metastasis were characterized by increased apelin serum level. Since we did not detect a positive correlation between tissue and serum apelin level, we wanted to know if there exists a positive relationship between apelin serum level and clinicopathological parameters, which could be the effect of increased BMI of CRC patient according to data which indicated that apelin serum level increases in obese patients [22]. The correlation between serum apelin level and BMI of CRC patients revealed no significant differences, suggesting that BMI of CRC patients had no influence on apelin serum level. Patient serum, as an easily accessible and valuable diagnostic tool, could be used to determine the stage of disease or survival through apelin level examination. However, this procedure has several misstatements. The presence of apelin in serum suggests that it is an endocrine peptide [22]. However, during blood vessel formation under normal and pathological conditions, apelin expression is associated with angiogenic blood vessel growth, where it acts through paracrine and autocrine mechanisms [27]. Therefore, the origin of apelin in serum is not clear. Moreover, the presence of various forms of apelin results in different activity. For example, apelin-13 and -17 have eight to 60-fold higher activity than apelin-36. Several studies showed differences in binding affinity (K_d_) and potency (EC_50_) for the different isoforms of apelin peptide [28]. In our study, we also showed the presence of the apelin receptor in CRC-patients serum using the ELISA technique. This method could detect free and membrane-bound receptors. Moreover, primary tumors, during progression, release into blood tumor-derived elements, such as circulating tumor cells, nucleic acids, exosomes and proteins [29]. Therefore, the upregulation of the apelin receptor during colorectal cancer progression is possible to detect in patients’ serum. Additionally, the role of peripheral blood in cancer diagnosis is still relevant. Recently, several studies focused on analysing circulating tumor-derived elements, which is called ‘liquid biopsy’. Traditional biopsy is an invasive procedure, especially if the tumor is located very deep, and it takes on risk of complications, including infection, bleeding, inflammation or even seeding tumour cells around the sampling area. Liquid biopsies represent a non-invasive, faster and safer alternative to tissue biopsy [29]. The identification of circulating tumor cells have been shown to have prognostic potential in different types of early-stage cancer, including colorectal cancer [30]. 

Summarizing, all these results suggest that apelin could be an important factor in colorectal cancer progression and it has the potential to be a prognostic factor, however further research connected with clinical endpoints is necessary. 

## 5. Conclusions

In summary, our studies indicate that apelin and its receptor expression is upregulated in primary colorectal cancer. Here, for the first time, we examined apelin and APJ mRNA, and protein tissue levels, that could be important clinical parameter in CRC disease. Moreover, apelin protein concentration correlated with APJ level in CRC individuals. Increased serum apelin levels corresponded with a higher TNM stage and the ability to develop lymph node and distant metastasis. Presented results suggest that apelin could be an important factor in the progression of colorectal carcinoma.

## Figures and Tables

**Table 1:** Distribution of demographical, clinical and pathological variables in patients with colorectal cancer (CRC). Data are presented as number of observations (percent of tested population) or mean ± SD.

Variable	CRC Patients (*n* = 56)
Gender:	
Female	24 (42.9)
Male	32 (57.1)
Age (years)	68.4 ± 11.1
Age ranges (years):	
<60	13 (23.2)
≥60	43 (76.8)
BMI (kg/m^2^)	27.5 ± 4.1
Disease stage (TNM):	
I	17 (30.4)
II	17 (30.4)
III	13 (23.2)
IV	9 (16.0)
Primary tumor progression (T):	
T1	5 (8.9)
T2	14 (25.0)
T3	33 (58.9)
T4	4 (7.1)
Lymph node metastasis (N):	
N0 (no regional lymph node metastasis)	34 (60.7)
N1 (regional lymph node metastasis)	22 (39.3)
Distant metastasis (M):	
M0 (no distant metastasis)	47 (83.9)
M1 (distant metastasis)	9 (16.1)
Differentiation:	
G2	53 (94.6)
G3	3 (5.4)
Tumor location:	
Right colon	20 (35.7)
Left colon	33 (58.9)
Rectum	3 (5.4)

**Table 2:** Apelin and apelin receptor mRNA expression levels in CRC patients. Gene expression was normalized to glyceraldehyde 3-phosphate dehydrogenase (GAPDH) and compared to non-tumor tissue using the Wilcoxon test. * Statistically significant at *p* < 0.05.

Gene Expression	Non-tumour tissue. mean ± SD median (min–max)	Tumour tissue. mean ± SD median (min–max)	Tumour/non-tumour ratio mean ± SD median (min–max)	*p*-Value
Apelin	0.003 ± 0.02 0.00 (0.00–0.13)	0.006 ± 0.02 0.00065 (0.00–0.049)	2.56 ± 6.51 0.00 (0.00–35.46)	0.0007 *
Apelin receptor	0.08 ± 0.15 0.03 (0.00–0.82)	0.11 ± 0.17 0.04 (0.00–0.76)	4.24 ± 8.25 1.64 (0.00–49.69)	0.03 *

**Table 3:** Apelin and apelin receptor tissue level in CRC patients. Tissue level of apelin and APJ was normalized to non-tumour tissue, and analyzed using the Wilcoxon test. * Statistically significant at *p* < 0.05.

Variable	Non-Tumour tissue. mean ± SD median (min–max)	Tumour tissue. mean ± SD median (min–max)	Tumour/non-Tumour ratio mean ± SD median (min–max)	*p*-Value
**Apelin (ng/g of tissue)**	36.00 ± 17.33 34.27 (6.05–101.6)	48.91 ± 18.30 47.77 (4.24–113.37)	1.68 ± 1.37 1.4 (0.21–8.44)	<0.0001 *
**Apelin receptor (μg/g of tissue)**	10.34 ± 13.07 5.29 (0.19–54.7)	32.32 ± 20.20 31.46 (1.11–72.35)	7.98 ± 9.59 5.58 (0.2–53.52)	<0.0001 *

## Supplementary Materials

The following are available online at https://www.mdpi.com/2077-0383/8/10/1513/s1, Figure S1: Exemplary amplification plot showing curves for APLN, APLNR and GAPDH genes; Figure S2: Correlation between serum and tissue apelin levels in CRC patients. These data were analysed using the Spearman’s rank correlation coefficient (ρ); Figure S3: Comparison of apelin receptor tissue level in men and women diagnosed with CRC. The data were analyzed using Mann-Whitney U test; Figure S4: Apelin tissue levels in patients with different stages of CRC. These data were analyzed using the post-hoc Dunn’s test; Figure S5: Apelin tissue levels in CRC patients without (N0) or with (N1) lymph node metastasis (**A**) or without (M0) or with (M1) distant metastasis (**B**) The data were analyzed using the Mann–Whitney U test, Figure S6: Correlation between apelin serum level and BMI in CRC patients. These data were analysed using the Spearman’s rank correlation coefficient (ρ).
