# Supplementary figures and images for: Evaluation of Apelin and Apelin Receptor Level in the Primary Tumor and Serum of Colorectal Cancer Patients
Source: J Clin Med. 2019 Sep 20;8(10):1513. doi: 10.3390/jcm8101513 (PMC6832595; doi:10.3390/jcm8101513)

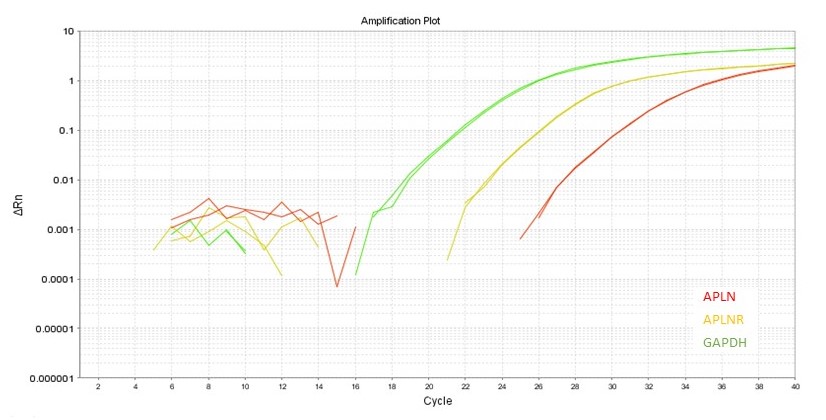

Supplement: Supplementary file 1 [file jcm-08-01513-s001.zip › jcm-573514-supplementary/Supplementary Material/Supplementary Figure 1.jpg]

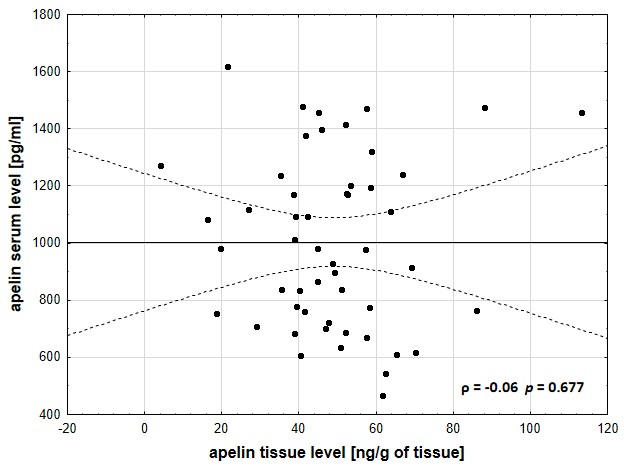

Supplement: Supplementary file 1 [file jcm-08-01513-s001.zip › jcm-573514-supplementary/Supplementary Material/Supplementary Figure 2.jpg]

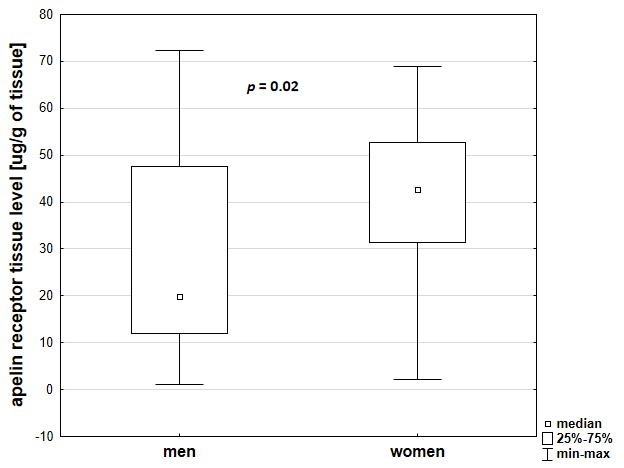

Supplement: Supplementary file 1 [file jcm-08-01513-s001.zip › jcm-573514-supplementary/Supplementary Material/Supplementary Figure 3.jpg]

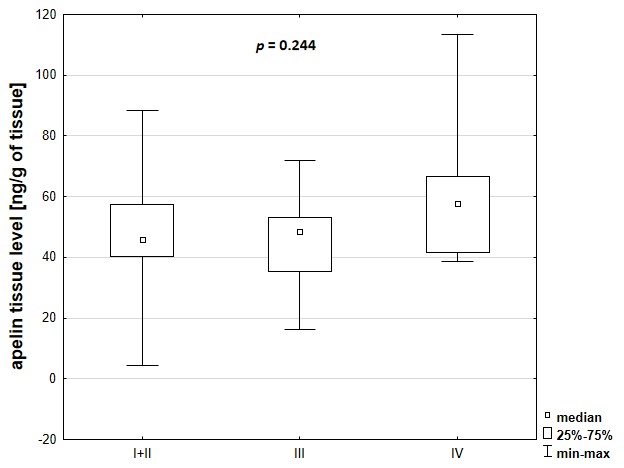

Supplement: Supplementary file 1 [file jcm-08-01513-s001.zip › jcm-573514-supplementary/Supplementary Material/Supplementary Figure 4.jpg]

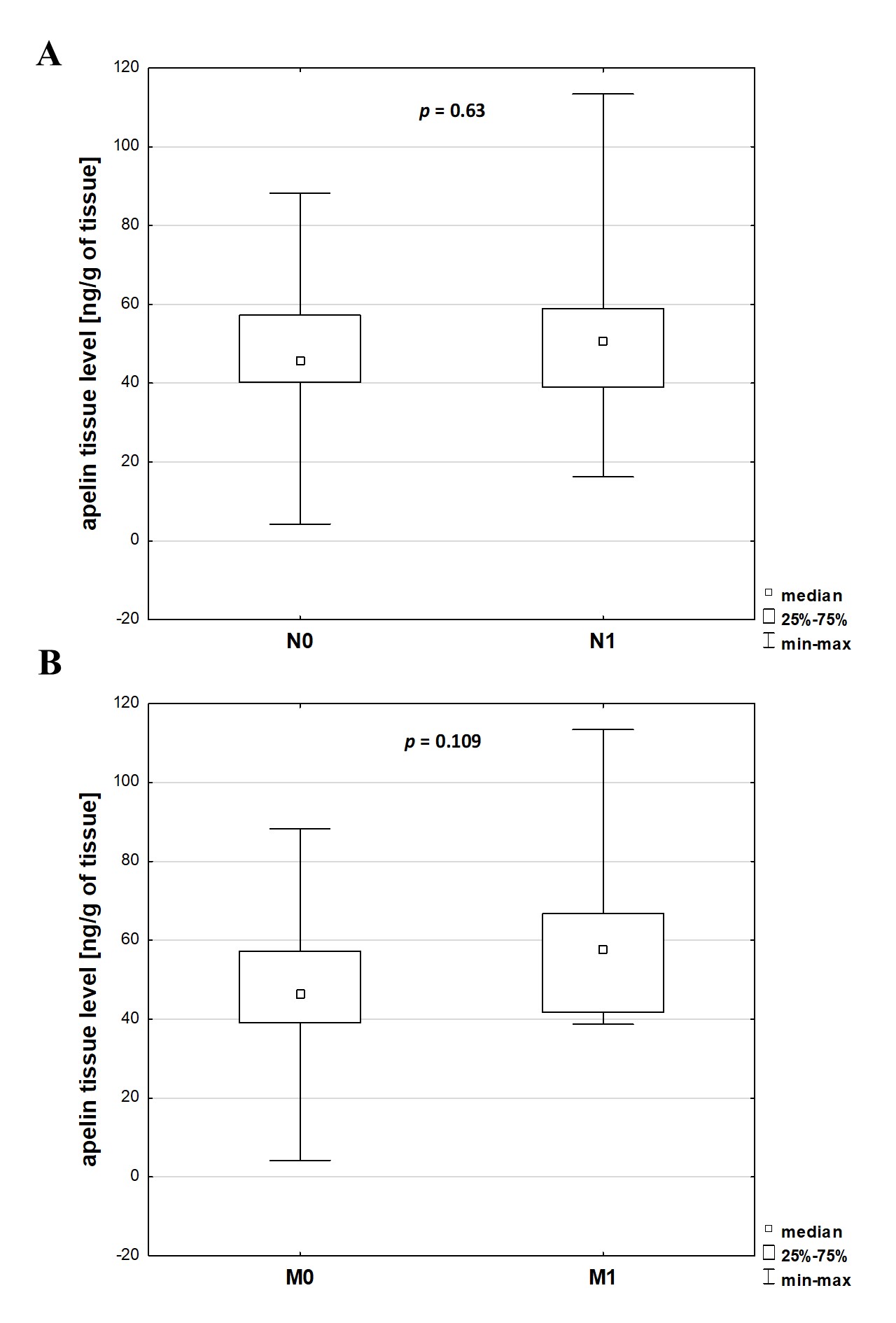

Supplement: Supplementary file 1 [file jcm-08-01513-s001.zip › jcm-573514-supplementary/Supplementary Material/Supplementary Figure 5.jpg]

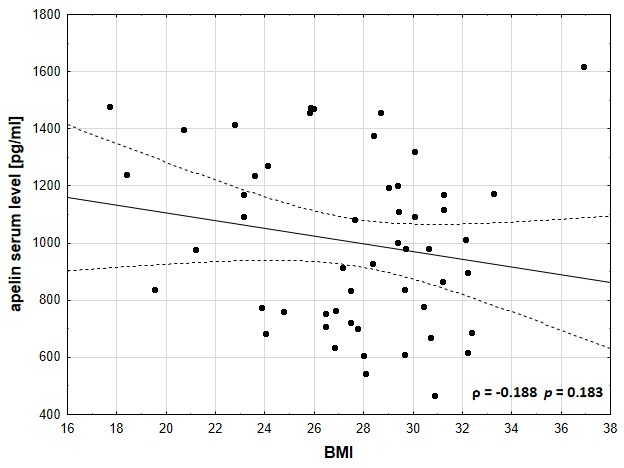

Supplement: Supplementary file 1 [file jcm-08-01513-s001.zip › jcm-573514-supplementary/Supplementary Material/Supplementary Figure 6.jpg]
